# Phenolic Profile, Fatty Acid Composition, and Antioxidant Activity of Italian Riesling Grape Pomace from Two Transylvanian Microclimates

**DOI:** 10.3390/plants14121809

**Published:** 2025-06-12

**Authors:** Veronica Sanda Chedea, Liliana Lucia Tomoiagă, Mariana Ropota, Gabriel Marc, Floricuta Ranga, Maria Comșa, Maria Doinița Muntean, Alexandra Doina Sîrbu, Ioana Sorina Giurca, Horia Silviu Răcoare, Corina Ioana Bocsan, Anca Dana Buzoianu, Hesham Kisher, Raluca Maria Pop

**Affiliations:** 1 Research Station for Viticulture and Enology Blaj (SCDVV Blaj), Gheorhe Barițiu Street, No 2, 515400 Blaj, Romania; tomoiagaliliana@yahoo.com (L.L.T.); comsa_m@yahoo.com (M.C.); maria.doinita@gmail.com (M.D.M.); sirbu.alexandra@ymail.com (A.D.S.); tirnovean.ioana@gmail.com (I.S.G.); racoarehoriasilviu@yahoo.com (H.S.R.); 2 Laboratory of Chemistry and Nutrition Physiology, National Research Development Institute for Animal Biology and Nutrition (IBNA Balotesti), Ilfov, 077015 Balotesti, Romania; m.ropota@yahoo.com; 3 Department of Pharmaceutical Chemistry, “Iuliu Hațieganu” University of Medicine and Pharmacy, 41 Victor Babeș Street, 400012 Cluj-Napoca, Romania; marc.gabriel@umfcluj.ro; 4 Food Science and Technology, Department of Food Science, University of Agricultural Science and Veterinary Medicine Cluj-Napoca, Calea Mănăștur, No 3-5, 400372 Cluj-Napoca, Romania; flori-cutza_ro@yahoo.com; 5 Pharmacology, Toxicology and Clinical Pharmacology, Department of Morphofunctional Sciences, “Iuliu Haţieganu” University of Medicine and Pharmacy, Victor Babeș, No 8, 400012 Cluj-Napoca, Romania; bocsan.corina@umfcluj.ro (C.I.B.); abuzoianu@umfcluj.ro (A.D.B.); 6 School of Applied Sciences, University of the West of England, Bristol BS16 1QY, UK; hesham.kisher@uwe.ac.uk

**Keywords:** Italian Riesling grape pomace, phenolics, fatty acids, antioxidant activity, microclimate

## Abstract

Italian Riesling is a grapevine (*Vitis vinifera*) cultivar widely grown in Transylvania vineyards. During the winemaking process, grape pomace (GP) is generated. This study aimed to exploit the potential of the Italian Riesling GP through its composition in polyphenols and fatty acids, as well as its antioxidant activity. Thus, two Italian Riesling GPs from two distinct Transylvanian microclimates (Crăciunelu de Jos and Ciumbrud) were analysed in terms of their phenolic and fatty acid composition and antioxidant activity while considering the influence of their respective microclimates. Every vineyard has unique geographical and meteorological characteristics that significantly influence grape production and consequently the structure of the resultant pomace. For example, Ciumbrud has a warmer, drier microclimate, whereas Crăciunelu de Jos has a colder, more humid environment. Biochemically, GP from Ciumbrud Italian Riesling grapes (RICI) contained greater amounts of gallic acid, total phenolic acids, and procyanidins and presented improved antioxidant activities, as reflected by DPPH˙, ABTS˙^+^, CUPRAC, and FRAP assays. RICI pomace also possessed a better fatty acid profile with higher oleic and linolenic acid levels, leading to a lower thrombogenicity index (TI) and a better PUFAω-6/PUFA ω-3 ratio. However, GP produced from Crăciunelu de Jos Italian Riesling grapes (RICR) possessed more catechin, epicatechin, epicatechin gallate, total flavanols, and higher COX values. The findings demonstrate that the two GPs have significant and distinct nutritional content, highlighting them as valuable resources for food consumption, providing benefits to consumers’ health.

## 1. Introduction

In Europe, Riesling has been grown since the Middle Ages [1]. In the 1800s, Riesling was brought to Italy, most likely from Germany’s Rhine Valley [2]. In Italy, Riesling is called Riesling renano [2]. Riesling Italico, sometimes called Walsch or Welsch Riesling, differs from Riesling Renan in morphology and wine production [2].

As reported by the Vitis International Variety Catalogue (VIVC) in 2008, Romania had the largest area planted with Riesling, with 7061 hectares (ha), followed by Hungary (4909 ha), Austria (3597 ha), Slovakia (3132 ha), Slovenia (2482 ha), Italy (2111 ha), and Brazil (1052 ha) [3,4]. Situated in central Transylvania, Alba county, Romania, Crăciunelu de Jos (part of the Târnave Vineyard), and Ciumbrud (part of the Aiud Vineyard) are two prominent viticultural regions known for their distinctive terroirs. With varying topographies, soil compositions, and geographical orientations, these vineyards present unique environmental conditions that significantly influence grape growth and wine quality. In both vineyards, the Italian Riesling cultivar is extensively cultivated, representing a significant proportion of the vineyard area [5]. This prevalence highlights the economic and agronomic importance of this cultivar in the region and justifies its selection for valorisation studies. Italian Riesling wine is a high-quality wine with a straw-yellow colour with greenish reflections. The pale-green hue reflects its freshness. The nose is fruity, and on the palate, it is fresh, dry, and medium-bodied [6].

During the winemaking process, grape pomace (GP) is a by-product that comprises 10%–30% of the mass of crushed grapes [7,8]. Specifically, approximately 20% of Italian Riesling grapes grown in Transylvania produce GP [9]. This includes pomace skins, pulp, stems, and seeds, amounting to 25–35 kg of GP for every 100 L of wine produced [10]. Variation in freshness and moisture content across different GPs [11], as well as cultivar and terroir [8,9], all affect the yield of pomace generation.

GP valorisation has been studied from different perspectives: animal nutrition [12,13,14,15], the food industry [16,17], cosmetics [18,19] and pharmacology [20,21,22]. These perspectives are further determined by its content in bioactive compounds, which is also influenced by the grapevine cultivar, terroir, and viticultural and wine production techniques [23,24]. The nutritional and bioactive substances found in GP are noteworthy and include carbohydrates (~12%–40%), fibres (~17%–88%), proteins (~4%–15%), lipids (~2%–14%), vitamins and minerals (~2%–7%), and polyphenols (~0.2%–9%) [24,25,26]. Thus, white GP, like that of Italian Riesling, contains high levels of polyphenols such as cinnamic acids (p-coumaric) and benzoic acids (syringic, gallic, protocatechuic, and 4-hydroxybenzoic), flavan-3-ols (catechin, epicatechin), proanthocyanidins, and flavonols (myricetin, quercetin, and kaempferol) [21,27]. According to reports, these substances exhibit various pharmacological properties, including anti-inflammatory, antifungal, antibacterial, and antioxidant behaviours [28,29,30,31,32]. Because phenolics can function as hydrogen donors, metal chelators, free-radical scavengers, and singlet-oxygen quenchers [29], their antioxidant capability is one of the most researched benefits for protecting human health.

A component of GP, the grape seed fraction, enriches it with fatty acids, most of which are unsaturated fatty acids [33]. Depending on the cultivar, GP contains mainly linoleic acid (C18:2), oleic acid (C18:1), and linolenic acid (C18:3) [33]. Linoleic and linolenic acids are essential for the human metabolic system, and as the human body cannot synthesize them, they must be consumed from external nutritional sources [33].

This study aimed to provide insights into Italian Riesling GP composition in terms of polyphenols and fatty acids, as well as its antioxidant activity, from two different Transylvanian microclimates.

## 2. Results

### 2.1. Total Polyphenol Content

The total polyphenol content of the two analysed samples was 74.75 ± 4.01 mg GAE/g GP for RICR and 67.01 ± 3.46 mg GAE/g GP for RICI, the difference being statistically significant.

### 2.2. FTIR Analysis of Grape Pomace Extracts

**Figure 1:**
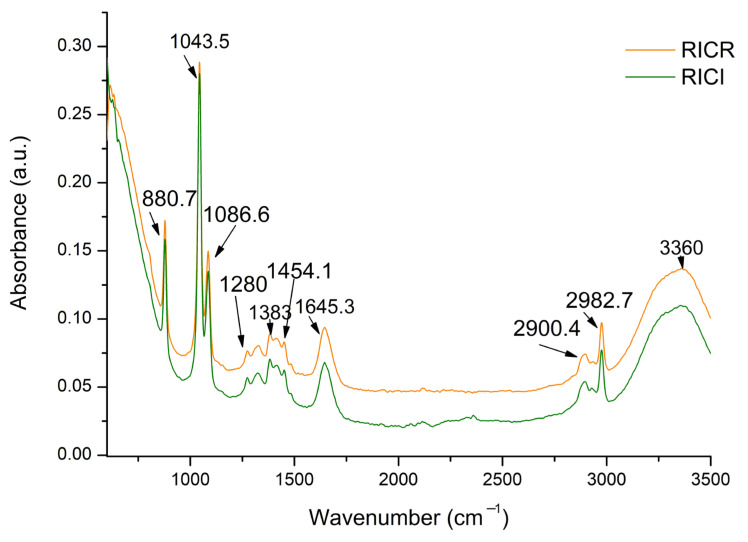
FTIR spectra of Crăciunelu de Jos Italian Riesling grape pomace (RICR) and Ciumbrud Italian Riesling grape pomace (RICI) recorded between 650 cm^−1^ and 3500 cm^−1^.

Figure 1 shows the FTIR spectra of the two studied GP extracts. The absorption bands align with those reported in similar studies [34,35,36,37,38,39].

The first broad band, having a maximum at 3360 cm^−1^ (Figure 1), is attributed to the phenolic groups having the hydroxyl group (−OH) bound to the aromatic hydrocarbon group [37,40]. In the 3500–3200 cm^−1^ range, the FTIR spectra generally indicate the occurrence of hydroxyl groups (−OH) [37,40]. This band assigned to the hydroxyl groups also indicates the presence of sugars [36,38], which remained in the grape pomace, especially in the white ones, because of the classical technology of making the white wines without the pomace maceration. The broad band of 3000 cm^−1^ to 3500 cm^−1^ of O−H stretching vibration was also linked to the NO−H stretching H bond stretching found in lignocellulosic components of the GP [39,41]. The absorption bands in the range of 2980–2900 cm^−1^ with the high-intensity peaks at 2982.7 cm^−1^ and 2900 cm^−1^ (Figure 1) can be assigned to the aliphatic −CH_3_ and –CH_2_ groups and symmetric and asymmetric C–H stretching [37,38,39,42,43]. These were attributed to the −CH_2_ asymmetric and symmetric lipids stretching vibrations [35] and the C−H in the lignin components [39,44]. The bands absorbing at 1645.3 cm^−1^, 1454.1 cm^−1^, and 1383 cm^−1^ (Figure 1) are linked to aromatic squeal vibration [38]. At 1645.3 cm^−1^, we can also associate the band with the asymmetrical and symmetrical −COO− stretching of the carboxylate compounds [37,42] like the hydroxybenzoic acids found in GP composition [21]. The peaks observed between 1320 cm^−1^ and 1460 cm^−1^ (Figure 1) are attributed to the non-symmetric scissoring and bending of CH_3_ from aliphatic compounds [45]. In the range of 1280 cm^−1^ and 1000 cm^−1^ (Figure 1), peaks were identified from the vibrations between C and O in the water-soluble components [38,39], such as polysaccharides [35] and C−6 of cellulose [39,41,46]. The sharp peak of 880.7 cm^−1^ (Figure 1) can be attributed to the C−H out-of-plane deformation in the pyranoside ring and mannose [37,40], as it is known that rhamnose, xylose, mannose, arabinose, galactose, glucose, and uronic acid represent the main monosaccharides in GP [47].

### 2.3. HPLC-DAD-ESI-MS Analysis of Grape Pomace Extract Polyphenols

Two phenolic acids, hydroxybenzoic and gallic acid, three procyanidins, procyanidin dimer B1, procyanidin dimer B2, procyanidin dimer B3, and four catechins, catechin, epicatechin, epicatechin gallate, and a catechin derivative, were identified in the two GPs (Table 1).

In terms of phenolic acids, RICI had statistically significantly higher values for gallic acid (30.35 ± 0.34 μg/mL) and total phenolic acids (137.57 ± 3.38 μg/mL) than RICR (22.19 ± 0.33 μg/mL for gallic acid and 122.98 ± 5.15 μg/mL for total phenolics, respectively) (Table 1). RICI also had higher concentrations of procyanidin dimer B1 (26.30 ± 0.2 μg/mL), procyanidin dimer B2 (33.56 ± 0.44 μg/mL), and procyanidin dimer B3 (35.05 ± 0.40 μg/mL) than RICR (23.41 ± 0.41 μg/mL for procyanidin dimer B1, 29.88 ± 0.40 μg/mL for procyanidin dimer B2, and 14.86 ± 0.37 μg/mL for procyanidin dimer B3, respectively) (Table 1). By contrast, RICR contained a higher amount of catechin (107.32 ± 4.98 μg/mL), epicatechin (142.62 ± 5.85 μg/mL), epicatechin gallate (26.58 ± 0.39 μg/mL), catechin derivate (328.68 ± 17.3 μg/mL), and by consequence total flavanols (673.34 ± 29.72 μg/mL) than RICI (63.25 ± 2.05 μg/mL for catechin, 87.38 ± 3.19 μg/mL for epicatechin, 20.17 ± 0.25 μg/mL for epicatechin gallate, 280.58 ± 8.40 μg/mL for catechin derivate and 546.29 ± 15.03 μg/mL for total flavanols, respectively) (Table 1).

The two identified flavonoids had a total concentration of 13.85 ± 0.24 μg/mL in RICI pomace with 12.34 ± 0.23 μg/mL of quercetin glucoside and 1.52 ± 0.00 μg/mL kaempferol glucoside (Table 1). RICR contained lower amounts of these compounds: 10.55 ± 0.44 μg/mL total flavonoids, 9.09 ± 0.43 μg/mL quercetin glucoside and 1.47 ± 0.01 μg/mL kaempferol glucoside (Table 1).

### 2.4. GC-FID Analysis of Fatty Acids

The different fatty acids that were detected and quantified in the investigated GPs are listed in Table 2: caprylic acid (C8:0), capric acid (C10:0) lauric acid (C12:0), myristic acid (C14:0), pentadecanoic acid (C15:0), palmitic acid (C16:0), heptadecanoic acid (C17:0), stearic acid (C18:0), arachidic acid (C20:0), lignoceric acid (C24:0), pentadecanoic acid (C15:1), palmitoleic acid (C16:1), oleic acid (C18:1), linolenic acid (C18:3n3), octadecatetraenoic (C18:4n3), eicosatrienoic acid (C20:3n3), eicosapentaenoic acid (C20:5n3), cis-linoleic acid (C18:2n6), eicosadienoic acid (C20:2n6), eicosatrienoic acid (C20:3n6), arachidonic acid (C20:4n6), and docosadienoic acid (C22:2n6).

Palmitic acid (C16:0) was the most abundant SFA, having equal concentrations in RICR (11.19 ± 0.05%) and RICI (11.16 ± 0.00%) (Table 2). In terms of total SFA, RICR (16.86 ± 0.07%) had a higher amount than RICI (16.23 ± 0.13%) (Table 2). Equal levels of UFA were found in both cultivars (82.94 ± 0.02% RICR vs. 82.96 ± 0.10% RICI) (Table 2). RICR had a significantly lower (17.47 ± 0.00%) concentration of MUFA than RICI (18.96 ± 0.05%) but a significantly higher PUFA (65.47 ± 0.02% RICR vs. 64.00 ± 0.05% RICI) (Table 2).

Linoleic acid was present in a concentration of 63.73 ± 0.03% for RICR and 61.18 ± 0.01% for RICI (Table 2). Oleic acid had values of 18.13 ± 0.02% for RICI and 16.55 ± 0.04% for RICR, significantly lower than RICI (Table 2). The linolenic acid concentration was 0.85 ± 0.01% for RICR and 1.87 ± 0.00% for RICI. Calculated oxidizability (COX), the ratio of fatty acids associated with hypocholesterolemic effects to those associated with hypercholesterolemic effects (H/H), unsaturation ratio (UFA/SFA), PUFA/SFA ratio, atherogenicity index (AI), and thrombogenicity index (TI) are also presented in Table 2.

The GPs studied presented COX values of 6.91 ± 0.00% for RICR and 6.75 ± 0.01% for RICI (Table 2). The UFA/SFA ratio for RICR and RICI is between 4.92 ± 0.02 and 5.11 ± 0.05, respectively, a difference that was not statistically important (Table 2). In Table 2, the same trend is observed for the PUFA/SFA ratio, RICR having the PUFA/SFA ratio of 3.88 ± 0.01 and RICI 3.94 ± 0.04 (Table 2). The PUFAω-6/PUFA ω-3 ratio reached values of 46.26 ± 0.89 for RICR and 29.07 ± 0.42 RICI (Table 2). No difference was observed for AI between RICI (0.15 ± 0.00) or RICR (0.15 ± 0.00), but RICR (0.35 ± 0.00) had a higher TI than RICI (0.34 ± 0.00) (Table 2).

### 2.5. Antioxidant Activity

To gain a complete image of the antioxidant potential of the pomace extracts, multiple assays involving different reagents were performed. Thus, the radical scavenging (ABTS˙^+^ and DPPH˙) and the electron transfer potential of the extracts (CUPRAC, FRAP and RP) were assessed. Because the free transition ions (especially Fe^2+^ and Cu^2+^) are involved in the generation of free radicals in organism, their potential chelation by the studied extracts was evaluated. This potential mechanism can act as a complementary antioxidant mechanism, associated with the main antioxidant effect of the extracts.

#### 2.5.1. Antiradical Potential

The extracts’ antiradical activity was assessed spectrophotometrically by measuring their capacity to scavenge DPPH˙ and ABTS˙^+^. The findings are presented in Table 3. RICI exhibited the strongest antiradical activity in both DPPH˙ and ABTS˙^+^ scavenging assays, with 88.61 ± 0.79 µM TE/mL extract for DPPH˙ and 3.00 ± 0.03 µM TE/mL extract for ABTS˙^+^ (Table 3).

#### 2.5.2. Electron Transfer Assays

Results of the electron transfer assays using the FRAP, RP, and CUPRAC methods, which were applied to the studied pomace extracts, are presented in Table 3. In the CUPRAC and FRAP assays, RICI had better activity than RICR: 1.08 ± 0.02 mg GAE/mL extract versus 0.89 ± 0.01 mg GAE/mL extract for CUPRAC and 1.81 ± 0.02 mg GAE/mL extract versus 1.54 ± 0.05 mg GAE/mL extract for FRAP (Table 3). In the RP test, both extracts reacted at the same level, 1.11 ± 0.03 mg GAE/mL extract for RICR and 1.11 ± 0.03 mg GAE/mL extract for RICI (Table 3).

#### 2.5.3. Metal Ion Chelation Assays

The results of the ferrous and cupric ion chelation assays are presented in Table 3. It was found that both RICR and RICI extracts exhibited an equal chelation effect, 0.23 ± 0.00 μM EE/mL extract and 0.22 ± 0.01 μM EE/mL extract, respectively, regarding chelation of ferrous ions (Table 3). The chelating capacity of pomace extracts on cupric ions was higher compared to that of ferrous ions. The extract with the highest chelation potential of the cupric ions was RICR with 0.41 ± 0.02 µM EE/mL (Table 3).

## 3. Discussion

Plants are dynamically influenced by their microclimate, as environmental factors like temperature, humidity, and radiation affect physiological processes, and in turn the plant’s presence can locally alter these microclimatic conditions [48]. A detailed understanding of these localised interactions enables viticulturists to make informed decisions regarding cultivar selection and vineyard management practices, ultimately improving grape and wine quality, and by extension the quality of GP [49].

Crăciunelu de Jos and Ciumbrud are situated within the historical Târnavelor and Aiud vineyards, respectively, and are among the oldest wine-producing regions in Transylvania [50,51]. The vineyards produce white, dry, and semi-dry wines [51].

Crăciunelu de Jos is situated at an altitude of ~235 m, and is located between the Lopadei Hills and the Secaselor Plateau. It has a moderate temperate continental climate with cold, humid winters (down to −24.7 °C) and warm summers (up to 41.6 °C) [50,52]. Autumn weather is typically clear and foggy, conditions that encourage slow grape ripening and the preservation of aromatic compounds [53]. The region accumulates significant thermal energy (3456.7 °C GHU/year), with July peaking at 655.7 °C and sunlight hours reaching 265 in June [52]. Annual rainfall averages 620 mm, predominantly falling in May and June, while humidity peaks during winter [52]. Soils include cambic and argic chernozems, marly phaeozems, and alluvial types, offering medium fertility [50].

Ciumbrud, located at the intersection of mountainous and plateau terrain, benefits from protective topography that shields it from harsh winds and creates favourable viticultural microclimates [51]. The region features varied elevations and slope gradients (8–18°), influencing solar radiation exposure and air circulation patterns [51]. The climate is moderately continental, with average annual temperatures of 9.3 °C (−3.5 °C in January to 20.3 °C in July) and frequent fog in late summer and autumn [54]. During ripening, daytime temperatures average 23 °C and night-time ~12 °C, contributing to balanced sugar accumulation and acidity retention [54]. Annual rainfall is ~615 mm, with optimal relative humidity (60–80%) for vine growth [54]. The dominant soils, brown luvic and alluvial types, are clayey and promote high-quality viticulture due to their moderate texture, humus content (2.5–4%), near-neutral pH, and balanced nutrient availability [54].

In summary, Crăciunelu de Jos is characterised by a cooler, more humid climate, while Ciumbrud experiences drier and slightly warmer conditions with superior solar exposure. These environmental differences are likely to influence the biochemical profiles of grapes and consequently the resulting pomace composition.

GP from RICR exhibited a higher total polyphenol content (TPC) compared to RICI. The TPC levels in Italian Riesling from Transylvania were consistent with those reported for Riesling GPs from other regions, including Moravia (47.94 mg/g) [55], Baden-Baden-Neuweier, Germany (50.94 g GAE/kg DM) [56], Bingen, Germany (44 mg GAE/g GP) [57], Teremia Mare, Romania (92.99 mg GAE/g GP) [58], Fruška Gora, Serbia—Bajilo (24.10 ± 0.13 mg GAE/g) and Agner (16.01 ± 0.43 mg GAE/g) [59], and Pietroasa-Istrița, Romania (24.00 mg GAE/g GP).

LC-MS analysis identified the presence of phenolic acids, flavanols, and flavonols in both RICR and RICI samples. RICI had higher gallic acid and total phenolic acids. Only two hydroxybenzoic acids—2-hydroxybenzoic and gallic—were detected in both, aligning with previous findings related to Muscat Ottonel grapes [60]. Hydroxycinnamic acids such as chlorogenic, caffeic, and ferric acids were also present [60]. In contrast, Spanish Moscatel grapes mainly contain caftaric and coutaric acids [61], while Chinese Riesling has been reported to contain only vanillic acid hexose ester [62]. Serbian Riesling samples demonstrated a broader spectrum, including gallic, protocatechuic, gentisic, chlorogenic, caffeic, and ferric acids across various grape tissues [63].

Flavan-3-ols dominated the phenolic profiles of both samples, accounting for 83.45% in RICR and 78.30% in RICI [59]. Variations in catechin and epicatechin levels have been associated with microclimate differences, as observed in Serbian Riesling GP [59]. RICR and RICI also contained procyanidin dimers B1–B3; however, RICI was richer in dimers and RICR higher in catechin, epicatechin, and total flavanols.

Regarding flavonol content, RICI showed higher levels of total flavonols, quercetin glucoside, and kaempferol glucoside compared to RICR, although these differences were not statistically significant. Quercetin was the predominant flavonol in both samples (RICR: 86.16%, RICI: 89.03%), followed by kaempferol (RICR: 13.84%, RICI: 10.97%). These distributions are consistent with those reported for Riesling grape skins from Xinjiang, China (quercetin 88.37%, kaempferol 11.63%) [62]. These align with findings published by Mattivi et al. (quercetin: 72.46–96.90%, kaempferol: 2.33–26.34%) and Castillo-Munoz et al. (quercetin: 60.8–90.7%, kaempferol: 8.8–38.3%, isorhamnetin: ~1.5%) [64,65]. In contrast, Blaj Muscat Ottonel skins had only 50.84% quercetin, 11.00% kaempferol, and 38.16% isorhamnetin. These compounds were absent in seeds [60]. Flavonols primarily accumulate in grape skins where they serve as protective agents against UVB radiation [66].

Given the established correlation between polyphenol content and antioxidant capacity, the antioxidant activity of RICR and RICI extracts was assessed through multiple mechanisms: radical scavenging, electron transfer, and metal chelation [67,68,69,70]. Due to the complexity of plant extracts, several assays were used [71,72,73,74], including DPPH˙ and ABTS˙^+^ assays. RICI demonstrated higher DPPH˙ and ABTS˙^+^ scavenging activity.

Electron-donating capacity was evaluated using FRAP, CUPRAC, and RP assays. FRAP (pH 3.6, RT) involves Fe^3+^ reduction with TPTZ complexation, producing a blue complex. RP (pH 6.6, 50 °C) reduces [Fe(CN)_6_]^3−^, forming Prussian blue. These assays differ in oxidant type and redox potential (FRAP: 0.77 eV; RP: 0.37 eV) [75,76]. In the CUPRAC assay, Cu^2+^ is reduced to Cu^+^, forming an orange complex with neocuproine. The extent of this reaction, indicating antioxidant activity, followed the same trend as FRAP—RICI showed higher electron transfer potential than RICR. However, measured gallic acid equivalents varied: highest in FRAP, followed by RP, and lowest in CUPRAC. Both pomaces showed comparably equal reducing power (RP), likely due to the energetic conditions of the assays favouring the detection of strong antioxidants while underrepresenting less reactive compounds.

The FRAP assay yielded significantly higher antioxidant values than the RP assay, an observation attributed to the FRAP assay involving a stronger oxidizing agent (Fe^3+^ vs. [Fe(CN)_6_]^3−^). This distinction emphasises and confirms that RP reflects the activity of highly potent antioxidants.

Chelation assays were used to evaluate the extracts’ ability to bind Fe^2+^ (using ferrozine) and Cu^2+^ (using murexide). Both pomaces exhibited similar Fe^2+^ chelation capacities. However, RICR demonstrated a notably stronger ability to chelate Cu^2+^.

As previously discussed, polyphenol presence is closely tied to antioxidant capacity. Compounds such as gallic acid, procyanidins, catechins, and flavonols contribute through mechanisms including hydrogen or electron donation and metal ion chelation [18,19,20,21,22,23,24,25,26,27].

Gallic acid, a trihydroxybenzoic acid, shows strong radical scavenging due to its three hydroxyl groups. Procyanidins and catechins enhance antioxidant capacity through their hydroxyl-rich structures, while flavonols like quercetin derivatives stabilize reactive oxygen and nitrogen species [18,19,20,21,22,23,24,25,26,27].

Among the samples, RICI consistently showed the highest antioxidant activity across almost all assays (DPPH, ABTS, FRAP, CUPRAC), correlating with its elevated levels of gallic acid and procyanidins. These findings support the superior antioxidant profile that RICI has.

FTIR analysis confirmed the presence of monosaccharides [47], lignocellulosic components [39,41], and lipids [35]. Grape seeds contributed significantly to the lipid faction, particularly bioactive fatty acids [47,77]. GC-FID identified 22 fatty acids in both RICR and RICI, comprising 10 saturated (SFA), 3 monounsaturated (MUFA), and 9 polyunsaturated (PUFA), exceeding previous reports [33,78].

RICR contained more SFAs, which are important for neuronal membrane function, but in excess, particularly palmitic acid (C16:0), have been associated with pro-inflammatory and metabolic disorders [47,78,79,80]. Prior studies confirm GP is low in SFAs (11–12%) and MUFAs (14–19%) and high in PUFAs (69–75%), especially linoleic acid [47,78,81,82,83,84].

Although both RCR and RICI exhibited identical total unsaturated fatty acid (UFA) content, their fatty acid compositions differed. RICR contained more polyunsaturated fatty acids (PUFAs), while RICI had more monounsaturated fatty acids (MUFAs), indicating terroir influences MUFA and PUFA distribution, but not total UFA content. These are consistent with Carmona-Jiménez et al. [33], who reported UFA levels of 83.41% in Spanish GPs (MUFA: 16.43–18.90%, PUFA: 64.04–66.87%).

Grape pomace fatty acids are predominantly unsaturated, with linoleic acids (60%) and oleic acids (18–20%), followed by palmitic (5–7%), stearic (3%), and myristic acids (3%) [47,85]. Linoleic acid was the dominant fatty acid in both samples, higher in RICR, consistent with Carmona-Jiménez et al. [33], who found 61.37–65.16%. This fatty acid aids in regulating LDL-C metabolism [47].

Oleic acid, the principal MUFA, was more abundant in RICI and fell within previously reported ranges (16.04–18.87%) [33]. Notably, linolenic acid, the primary ω-3 PUFA, was more than twice as concentrated in RICI than RICR. GP oil typically contains more linolenic acid (0.90–4.30%) than typical grape seed oil, with Ferreira et al. [78] reporting 1.94 ± 0.32%. This makes GP oil richer in linolenic acid than most vegetable oils, and only soy and rapeseed can reach comparable levels of up to 10% [33,78,86,87].

Overall, the fatty acid profile of GP is comparable to oilseeds like sunflower, corn, and soy, though it typically has lower linolenic acid levels [47,85].

Due to their high fatty acid content, GP oils are more susceptible to oxidation [33,88]. To assess this, the COX values were used as an indicator of oxidative stability of GP fatty acids. RICR exhibited a higher COX value than RICI, consistent with the findings published by Carmona-Jiménez et al. [33].

Fatty acid composition also reflects the functional quality of oils derived from by-products generated during the winemaking process. The unsaturated-to-saturated fatty acid (UFA/SFA) ratio was similar in both RICR and RICI, aligning with values reported for Spanish (4.78–6.04) and Portuguese (5.55) GPs [33,78].

The PUFA/SFA ratio is a key nutritional indicator of dietary fat quality, with values above 0.45 recommended for a healthy diet [33,89]. Both pomaces exceeded this threshold, reinforcing their potential as sources of health-promoting lipids.

GP oils typically show a high PUFAω-6/PUFAω-3 ratio [47,85]. RICI had a significantly lower and more favourable ratio than RICR. For comparison, Ferreira et al. [78] reported a ratio of 37.3 in Portuguese GP, higher than RICI but lower than RICR. Balancing PUFAω-6 and PUFAω-3 intake is vital for health, yet Western diets favour ω-6-rich oils, causing imbalance [33,86]. Oils derived from whole GP, not just seeds, tend to have higher linolenic acid content and thus yield a more favourable ω-6/ω-3 ratio [33,90].

The atherogenicity index (AI), thrombogenicity index (TI), and the hypo/hypercholesterolemic fatty acid ratio (H/H) are key indicators used to evaluate the cardiovascular health impact of dietary fats [33]. Both RICI and RICR had the same AI, but RICR exhibited a higher TI, indicating a slightly greater risk of thrombogenicity [78,91,92]. Elevated AI and TI values are associated with increased cardiovascular risk [78].

The H/H index was nearly equal in both GPs, with RICI showing a slightly lower value. Nutritionally, a higher H/H value is preferable, as it indicates a more favourable lipid profile for better cholesterol metabolism [78,83]. Although lower than linseed (13.24) [93], Spanish (10.54) [33], and Serbian grape seed oils (11.07–12.28) [83], both GPs had higher H/H values than olive oil (6.14) [93].

Spanish GPs typically exhibit AI values between 0.11–0.16 and TI values of 0.30–0.35, and Portuguese GP show similar values (AI: 0.11, TI: 0.30) [33,78]. Serbian grape seed oils have even lower indices (AI: 0.08–0.09, TI: 0.24–0.27) [83]. The H/H values for RICR (7.12) and RICI (7.08) fall within the range reported for Spanish GP (6.93–9.45) [33].

The UFA/SFA ratio and PUFAω-6/PUFAω-3 ratio are also crucial nutritional indicators. Low UFA/SFA and high ω-6/ω-3 ratios are considered harmful, contributing to higher cholesterol and obesity risks [78,94,95]. While both pomaces had similar AI, H/H, and UFA/SFA ratios, RICI exhibited a lower TI and ω-6/ω-3 ratio, suggesting a slightly better overall nutritional profile.

## 4. Materials and Methods

### 4.1. Chemicals

Acetonitrile (HPLC grade), acetic acid, ethanol, methanol (MS grade), hexane, petroleum ether, copper(II) chloride (2,2-CuCl_2_), neocuproine (2,9-dimethyl-1,10-phenanthroline, C_14_H_12_N_2_), potassium hexacyanoferrate (III) (K_3_[Fe(CN)_6_]),trichloroacetic acid (Cl_3_CCOOH), iron(III) chloride (FeCl_3_), ferrozine (3-(2-Pyridyl)-5,6-diphenyl-1,2,4-triazine-p,p′-disulfonic acid monosodium salt hydrate, C_20_H_13_N_4_NaO_6_S_2_·xH_2_O), murexide (5,5′-nitrilodibarbituric acid monoammonium salt, C_8_H_8_N_6_O_60_), and Supelco 37 Component FAME Mix were purchased from Merck (Darmstadt, Germany). Folin–Ciocâlteu reagent, diphenyl-1-picrylhydrazyl (DPPH), Trolox, ABTS˙^+^ (2,2′-azinobis-(3-ethylbenzthiazolin-6-sulfonic acid)), FRAP reagent, gallic acid, catechin, and quercetin (of 99% HPLC grade) were bought from Sigma Co. (St. Louis, MO, USA). Sulfuric acid (H_2_SO_4_), sodium sulphate anhydrous (Na_2_SO_4_), and sodium carbonate (Na_2_CO_3_) were obtained from Amex (Bucharest, Romania).

### 4.2. Grape Pomace Generation and Conditioning

**Figure 2:**
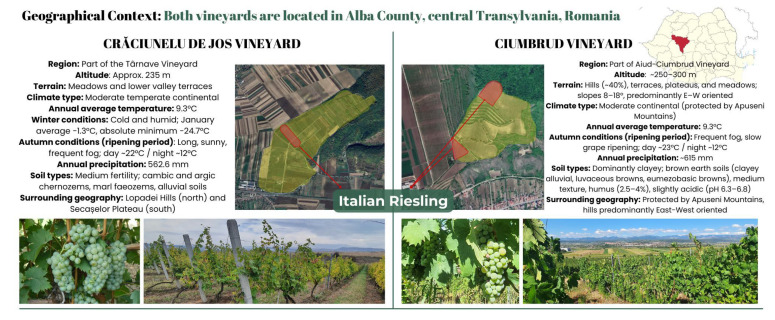
Geographical context of the two studied GPs (RICR and RICI).

The Research and Development Station for Viticulture and Winemaking Blaj (SCDVV Blaj) (Blaj, Târnave Wine Centre, Romania) provided the GPs of white wine grapes (*Vitis vinifera* L.) of the Italian Riesling cultivar. Between 12 September and 18 September 2019, the grapes were picked from the Ciumbrud vineyard (RICI) and the Crăciunelu de Jos vineyard (RICR) (Figure 2). Following grape pressing, the resultant GP (comprising stems, skins, pulp, and seeds) was collected, air-dried in a well-ventilated chamber at room temperature, and stored in paper bags in a dark room until extraction.

### 4.3. Grape Pomace Polyphenol Extraction

The raw grape pomace material was ground in a Cyclone Mill-MC5 (Tecator, Höganäs, Sweden) until the particles were 1 mm. A 3 mL solvent mixture of water and ethanol (30:70, *v*/*v*) was used to extract 0.1 g of GP powder. The GP extracts RICR and RICI were obtained by stirring the mixture for two hours at 600 rpm and filtering it through Whatman filter paper.

### 4.4. Total Polyphenol Content (TPC) of Grape Pomace Polyphenol Extracts

As previously stated, the Folin–Ciocâlteu method was used to determine the RICR and RICI extracts’ total polyphenol content [37]. Consequently, 25 μL of each GP extract was mixed with 100 μL sodium carbonate solution (7.5% *w*/*v*) and 125 μL of 0.2 N Folin–Ciocâlteu reagent. The mixture was further homogenised and then incubated for two hours at 25 °C in the absence of light. At 760 nm, the absorbance was measured using a Synergy HT Multi-Detection Microplate Reader (BioTek Instruments, Inc., Winooski, VT, USA). Gallic acid was utilised to create the calibration curve (R^2^ = 0.9945). Gallic acid equivalents (GAEs) were used to calculate the averages and standard deviations of the data, which are presented as follows: mg GAE/g dry weight (dw) GP (*n* = 3).

### 4.5. Fourier Transform Infrared Spectroscopy (FTIR) Analysis

A Shimadzu IR Prestige-21 spectrophotometer (Shimadzu Handelsgesellschaft mbH, Bucharest, Romania) equipped with a horizontal ATR (attenuated total reflectance) diamond accessory with single reflection (PIKE Technologies, Fitchburg, WI, USA) was used to perform FTIR analysis on the RICR and RICI pomace extracts. The background was methanol. At a resolution of 4 cm^−1^, the spectra were recorded between 4000 and 600 cm^−1^. In sum, 64 scans were taken for every spectrum. The IR solution software Overview, version 1.30 (Shimadzu, Northampton, Handelsgesellschaft mbH, Bucharest, Romania) and Origin^®^ 7SR1 Software (OriginLab Corporation, Northampton, MA, USA) were used to identify the absorption bands for various bond and functional group types and to analyse further the primary data that was obtained.

### 4.6. Liquid Chromatography–Diode Array Detection–Electrospray Ionisation Mass Spectrometry (HPLC-DAD-ESI MS) Analysis

As previously reported by Pop et al. [35] and Chedea et al. [60], the phenolic composition of the grape pomace extracts has been measured both qualitatively and quantitatively using an Agilent 1200 HPLC with a DAD detector in conjunction with an Agilent 6110 single quadrupole MS system. Following that, two mobile phases, A (0.1% acetic acid in distilled water (*v*/*v*)) and B (0.1% acetic acid in acetonitrile (*v*/*v*)), were run for 30 min at 25° C with a flow rate of 0.5 mL/min to separate the compounds using an Eclipse XDB C18 column (4.6 × 150 mm, particle size 5 μm, Agilent Technologies, Santa Clara, CA, USA). Gradient elution was performed as follows: 0–2 min, 5% B; 2–18 min, linear gradient from 5% to 40% B; 18–20 min, linear gradient from 40% to 90% B; 20–24 min, 90% B; 24–25 min, linear gradient from 90% to 5% B; 25–30 min, 5% B (where B represents the organic solvent). All UV-vis spectra were registered in the 200–600 nm wavelength range, and the chromatograms were registered at λ = 280, 340, and 520 nm. The analysis was carried out three times for each extract. At 3000 C, 7 L/min of nitrogen flow, and 3000 V capillary voltage, the ESI+ MS analysis was carried out. The entire scan was set between 100 and 1200 *m*/*z*. Agilent ChemStation software (Rev B.04.02 SP1, Palo Alto, CA, USA) was used to acquire chromatograms, spectra, and their analysis. Mass and UV-vis spectra and retention time were used to identify the phenols. Calibration curves of several reference solutions of rutin (R^2^ = 0.9981; LOD = 0.21 μg/mL, LOQ = 0.84 μg/mL), gallic acid (R^2^ = 0.9978; LOD = 0.36 μg/mL, LOQ = 1.44 μg/mL), and catechin (R^2^ = 0.9985; LOD = 0.18 μg/mL, LOQ = 0.72 μg/mL) were used for quantification. The quantification of hydroxybenzoic acids was done as gallic acid equivalents, flavanols as catechin equivalents, and flavonols as quercetin equivalents.

### 4.7. Gas Chromatography with Flame Ionisation Detector (GC-FID) Analysis of Fatty Acids

The dried GP’s total lipids were extracted in petroleum ether using a Foss Soxtec 2055 (Effretikon, Switzerland) system. Fatty acids from the total lipid extracts were transesterified to their methyl esters (FAME) in methanol with 3% concentrated sulphuric acid for four hours at 80 °C. One microlitre of FAME was injected into a Perkin Elmer Clarus 500 chromatograph (Bucharest, Romania) that was outfitted with a flame ionisation detector (FID) and a BPX70 capillary column (60 m × 0.25 mm i.d., 0.25 μm film thickness). Air oxygen burned at a rate of 420 mL min^−1^, the splitting ratio was 1:100, and the carrier gas was hydrogen, which flowed at a rate of 35 cm s^−1^ at 180 °C. The injector and detector temperatures were 250 °C and 260 °C, respectively, while the column temperature was adjusted between 180 °C and 220 °C by 5 °C. The length of the chain, the degree of unsaturation, and the double-bond geometry were used to differentiate FAME. The examined sample (or batch of samples) was analysed concurrently with a reference sample (Supelco 37 Component FAME Mix) and a control sample (n-hexane) [96]. The data are presented as grams of fatty acid per 100 g total fatty acids, and FAME identification was accomplished by comparing retention times to established standards. Every measurement was carried out three times (*n* = 3).

The calculated oxidizability (COX) values, atherogenicity index (AI), thrombogenicity index (TI), and the ratio between hypo- and hypercholesterolemic fatty acids (H/H) were calculated according to a previous study [33].

### 4.8. Antioxidant Activity

#### 4.8.1. Antiradical Assays

##### Assessment of Relative DPPH Radical-Scavenging Ability

Using the 2,2-diphenyl-1-picrylhydrazyl (DPPH) test, the radical-scavenging capacity of each grape pomace extract (RICR and RICI) was determined following the procedure described by Pop et al. [35]. A mixture of 1750 μL DPPH solution (0.02 mg/mL in methanol) and 250 μL of each GP extract was incubated for 30 min at room temperature. To measure absorbance at 517 nm, a Synergy HT Multi-Detection Microplate Reader (BioTek Instruments, Inc., Winooski, VT, USA) was employed. Computation of the calibration curve (R^2^ = 0.9985) and results was performed using Trolox (µM trolox/mL extract). The DPPH˙ radical-scavenging activity of GP extracts was calculated using Equation (1) and expressed as means ± standard deviations (*n* = 3):

(1) $$DPPH\;scavenging\;\left[\%\right]=\frac{control\;absorbance-sample\;absorbance}{control\;absorbance}\;\times \;100$$

##### ABTS Cation Radical-Scavenging Capacity Measurement (ABTS)

The ABTS˙^+^ (2,2′-azinobis-(3-ethylbenzthiazolin-6-sulfonic acid)) radical-scavenging assay was carried out according to a previously published procedure [97,98,99]. In short, 3900 µL of ABTS˙^+^ reagent solution and 10 µL of the 1:4 methanol diluted GP extracts were combined with 90 µL of methanol. At λ = 734 nm, the resulting solutions’ decrease in absorbance was measured spectrophotometrically (UV-vis Jasco V-530 spectrophotometer, Jasco International Co., Tokyo, Japan) in comparison to a blank sample consisting of 3900 µL of reagent solution and 100 µL of methanol. The ABTS scavenging activity of the GP extracts was calculated using Equation (2):

(2) $$ABTS\;scavenging\;\left[\%\right]=\frac{control\;absorbance-sample\;absorbance}{control\;absorbance}\;\times \;100$$

Computation of the calibration curve (R^2^ = 0.9985) and results was performed using gallic acid, expressed in milligrams of gallic acid equivalents/mL extract (mgGAE/mL) as means ± standard deviations (*n* = 3).

#### 4.8.2. Electron Transfer Assays

##### Measurement of Cupric Reducing Antioxidant Capacity (CUPRAC)

When performing the CUPRAC assay, 50 µL of the 1:4 methanol diluted GP extracts was mixed with 1000 µL CuCl_2_ 0.01 M, 1000 µL neocuproine 7.5 mM, and 1000 µL ammonium acetate buffer 1 M and shaken well for half an hour in the dark to yield an orange complex with absorption maximum at λ = 450 nm (UV-vis Jasco V-530 spectrophotometer, Jasco International Co., Tokyo, Japan). Computation of the calibration curve (R^2^ = 0.9985) and results was performed using gallic acid, expressed in milligrams of gallic acid equivalents/mL extract (mgGAE/mL) as means ± standard deviations (*n* = 3).

##### Measurement of Ferric Reducing Antioxidant Potential (FRAP)

The FRAP assay was performed using a modified method proposed initially by Benzie and Strain [100]. A measure of 10 µL from the 1:4 methanol-diluted GP extracts was mixed with 2000 μL 0.3 M acetate buffer (pH = 3.6) and 1000 μL of FRAP reagent. The absorbance of the samples was determined at λ = 593 nm (UV-vis Jasco V-530 spectrophotometer, Jasco International Co., Tokyo, Japan). Computation of the calibration curve and results was performed using gallic acid (R^2^ = 0.9985) and Trolox (R^2^ = 0.9985), expressed in milligrams of gallic acid equivalents/mL extract (mgGAE/mL) and micromolar Trolox equivalents/mL extract (µM TE/mL) as means ± standard deviations (*n* = 3).

##### Measurement of Reducing Power (RP)

For the RP assay, 10 µL of GP extract (diluted 1:4 in methanol) was mixed with 400 µL of phosphate buffer (0.2 M, pH = 6.6) and 400 µL of K_3_[Fe(CN)_6_] solution (1% *w*/*v*) in test tubes, which were sealed and placed in a water bath at 50 °C for 20 min. After cooling the test tubes, the reaction was stopped with trichloroacetic acid. In the final step, FeCl_3_ was added to yield a Perl’s Prussian blue complex with an absorption maximum at λ = 593 nm UV-vis (Jasco V-530 spectrophotometer, Jasco International Co., Tokyo, Japan). Computation of the calibration curve and results was performed using gallic acid (R^2^ = 0.9985), expressed in milligrams of gallic acid equivalents/mL extract (mg GAEs/mL) as means ± standard deviations (*n* = 3).

#### 4.8.3. Ferrous and Cupric Chelation Assays

The chelation potential of GP extracts for the ferrous and cupric ions was determined spectrophotometrically (Jasco V-530 spectrophotometer, Jasco International Co., Tokyo, Japan) using an adaptation of previously reported protocols [97,98,99]. The presence of a metal chelator in the environment would decrease the absorbance of the sample due to the disruption of the chromogenic complex.

The free ferrous ions in the solution created a red complex with ferrozine. For the measurement of the ferrous chelation activity, previously reported by Dinis et al. [101], the assay involved the mixture of 200 µL of the extracts, 500 µL of FeSO_4_ (0.125 mM), and 500 µL of ferrozine 0.315 mM. After 10 min, the absorbance of the solutions was measured at λ = 562 nm against a blank sample. The absorbance of the sample is proportional to the amount of complex formed, which in turn is proportional to the amount of free ferrous ions in the solution.

Similarly, murexide forms a complex with cupric ions, giving a strong purple complex. For the measurement of the copper chelation activity, previously reported by Wu et al. [102], the assay involved the mixture of 100 µL of the extracts and 400 µL of 3 mM CuSO_4_ in hexamine buffer (10 mM hexamine and 10 mM KCl), and after 5 min, 75 µL of murexide 1 mM and 2 mL of distilled water were added. After 5 min of incubation at room temperature, the absorbance was measured at λ = 485 and λ = 520 nm, because free murexide has its own colour with a maximum of absorption at λ = 520 nm, which was taken into account as presented [103].

Computation of the calibration curve and results was performed using EDTA (R^2^ = 0.9985), expressed in micromolar EDTA equivalents/mL extract (µM EEs/mL) as means ± standard deviations (*n* = 3).

### 4.9. Statistical Analysis

Data analysis was performed using the software IBM SPSS Statistics version 20 (SPSS Inc., Chicago, IL, USA). Independent-sample *t*-tests were conducted to compare the means of the dependent variables between the two groups (RICR and RICI). The assumption of homogeneity of variance was assessed using Levene’s test. If Levene’s test was significant (*p* < 0.05), indicating unequal variances, the *t*-test results were interpreted using the adjusted degrees of freedom.

## 5. Conclusions

The climate conditions in the Transylvanian region are generally favourable for grapevine growth, providing intense biological activity during the warm season. However, specific vineyard characteristics, such as elevation, soil composition and local weather patterns, lead to significant differences between Ciumbrud and Crăciunelu de Jos GP composition. The specific geographical and climatic conditions of each location—with Ciumbrud presenting a warmer and drier microclimate and Crăciunelu de Jos a cooler, more humid climate—profoundly impact grape development and consequently pomace composition. Biochemically, RICI pomace exhibited higher contents of gallic acid, total phenolic acids, and procyanidins, and higher antioxidant activities, revealed by DPPH˙, ABTS˙^+^, CUPRAC, and FRAP assays. RICI pomace also contained a more favourable fatty acid profile with greater oleic and linolenic acid content, which accounted for a lower thrombogenicity index (TI) and more favourable PUFAω-6/PUFA ω-3 ratio. RICR pomace, on the other hand, contained greater catechin, epicatechin, epicatechin gallate, and total flavanol contents and greater COX value. Terroir-specific factors impacted MUFA and PUFA content, though not significantly.

However, both pomaces are nutritionally significant, suggesting their potential as valuable commodities for food and health-related applications. Further research is warranted into the long-term effects of such regional variations on grape pomace composition and uses.

## Figures and Tables

**Table 1:** Phenolic acids (μg/mL gallic acid equivalents), flavanols (μg/mL catechin equivalents), and flavonols (μg/mL quercetin equivalents) quantities were determined in the grape pomace extracts by LC-MS.

Tentative Identification					Concentration ^†^ (μg/mL)	
Retention Time Rt (min)	UV λmax (nm)	[M + H]+ (m/z)	Compounds	Subclass	RICR	RICI
3.29	270	138	2-Hydroxybenzoic acid	Hydroxybenzoic acid	100.78 ± 4.81 ^a^	107.22 ± 3.03 ^a^
5.96	279	171	Gallic acid	Hydroxybenzoic acid	22.19 ± 0.33 ^a^	30.35 ± 0.34 ^b^
Total phenolic acids					122.98 ± 5.15 ^a^	137.57 ± 3.38 ^b^
10.42	280	579, 291	Procyanidin dimer B3	Flavanol	23.41 ± 0.41 ^a^	26.30 ± 0.29 ^b^
11.63	280	579, 291	Procyanidin dimer B1	Flavanol	29.88 ± 0.40 ^a^	33.56 ± 0.44 ^b^
12.28	280	579, 291	Procyanidin dimer B2	Flavanol	14.86 ± 0.37 ^a^	35.05 ± 0.40 ^b^
12.61	280	291	Catechin	Flavanol	107.32 ± 4.98 ^a^	63.25 ± 2.05 ^b^
13.95	280	291	Epicatechin	Flavanol	142.62 ± 5.85 ^a^	87.38 ± 3.19 ^b^
16.40	280	443	Epicatechingallate	Flavanol	26.58 ± 0.39 ^a^	20.17 ± 0.25 ^b^
22.91	280	n.d	Catechin derivate	Flavanol	328.68 ± 17.34 ^a^	280.58 ± 8.40 ^b^
Total flavanols					673.34 ± 29.72 ^a^	546.29 ± 15.03 ^b^
16.23	355	465	Quercetin-glucoside	Flavonol	9.09 ± 0.43 ^a^	12.34 ± 0.23 ^b^
17.33	253, 350	449	Kaempferol-glucoside	Flavonol	1.47 ± 0.01 ^a^	1.52 ± 0.00 ^b^
Total flavonols					10.55 ± 0.44 ^a^	13.85 ± 0.24 ^b^

^†^ Values represent mean triplicate ± standard deviation values and are expressed as μg mL^−1^ residue. The data were analysed using independent-sample *t*-tests, and within each row, different letters indicate statistical differences at *p* < 0.05; n.d.—not detected

**Table 2:** Fatty acid composition (g/100 g of dried sample ± standard deviation) of the studied grape pomace, determined using GC-FID.

Sample/Fatty Acid (g FAME/100 g Total FAME)	RICR	RICI
Saturated Fatty Acids (SFAs)		
Caprylic (C 8:0)	0.53 ± 0.04 ^a^	0.03 ± 0.02 ^b^
Capric (C 10:0)	0.09 ± 0.01 ^a^	0.06 ± 0.01 ^a^
Lauric (C12:0)	0.02 ± 0.00 ^a^	0.03 ± 0.02 ^a^
Myristic (C 14:0)	0.22 ± 0.02 ^a^	0.22 ± 0.05 ^a^
Pentadecanoic (C15:0)	0.06 ± 0.02 ^a^	0.04 ± 0.01 ^a^
Palmitic (C16:0)	11.19 ± 0.05 ^a^	11.16 ± 0.00 ^a^
Heptadecanoic (C17:0)	0.04 ± 0.01 ^a^	0.09 ± 0.01 ^a^
Stearic (C18:0)	4.19 ± 0.00 ^a^	4.32 ± 0.04 ^a^
Arachidic (C 20:0)	0.34 ± 0.03 ^a^	0.07 ± 0.02 ^b^
Lignoceric (C 24:0)	0.18 ± 0.00 ^a^	0.19 ± 0.03 ^a^
Total SFAs	16.86 ± 0.07 ^a^	16.23 ± 0.13 ^b^
Unsaturated Fatty Acids (UFAs)		
Monounsaturated Fatty Acids (MUFAs)		
Pentadecanoic (C15:1)	0.05 ± 0.01 ^a^	0.08 ± 0.01 ^a^
Palmitoleic (C16:1)	0.87 ± 0.03 ^a^	0.76 ± 0.04 ^a^
Oleic cis (C18:1)	16.55 ± 0.04 ^a^	18.13 ± 0.02 ^b^
Total MUFAs	17.47 ± 0.00 ^a^	18.96 ± 0.05 ^b^
Polyunsaturated Fatty Acids (PUFAs)		
Polyunsaturated Fatty Acid Omega 3 (PUFA ω-3)		
Linolenic (C18:3n3)	0.85 ± 0.01 ^a^	1.26 ± 0.01 ^b^
Octadecatetraenoic (C18:4n3)	0.36 ± 0.02 ^a^	0.69 ± 0.02 ^b^
Eicosatrienoic (C20(3n3))	0.07 ± 0.00 ^a^	0.07 ± 0.01 ^a^
Eicosapentaenoic (C 20:5n3)	0.10 ± 0.00 ^a^	0.12 ± 0.02 ^a^
Total PUFA ω-3	1.39 ± 0.03 ^a^	2.13 ± 0.03 ^b^
Polyunsaturated Fatty Acid Omega 6 (PUFA ω-6)		
Linoleic cis (C 18:2n6)	63.73 ± 0.03 ^a^	61.18 ± 0.06 ^b^
Eicosadienoic (C20(2n6))	0.12 ± 0.00 ^a^	0.00 ± 0.00 ^b^
Eicosatrienoic (C20(3n6))	0.05 ± 0.01 ^a^	0.07 ± 0.01 ^a^
Arachidonic (C20(4n6))	0.08 ± 0.00 ^a^	0.27 ± 0.01 ^b^
Docosadienoic (C 22:2n6)	0.11 ± 0.01 ^a^	0.36 ± 0.01 ^b^
Total PUFA ω-6	64.08 ± 0.04 ^a^	61.87 ± 0.02 ^b^
Total PUFA	65.47 ± 0.02 ^a^	64.00 ± 0.05 ^b^
Total UFA (MUFA+PUFA)	82.94 ± 0.02 ^a^	82.96 ± 0.10 ^a^
Other fatty acids	0.20 ± 0.09 ^a^	0.81 ± 0.04 ^b^
SFA/UFA	0.20 ± 0.00 ^a^	0.20 ± 0.00 ^a^
PUFA/MUFA	3.75 ± 0.00 ^a^	3.38 ± 0.01 ^b^
ω-6/ω-3	46.26 ± 0.89 ^a^	29.07 ± 0.42 ^b^
UFA/SFA	4.92 ± 0.02 ^a^	5.11 ± 0.05 ^a^
PUFA/SFA	3.88 ± 0.01 ^a^	3.94 ± 0.04 ^a^
COX	6.91±0.00 ^a^	6.75 ± 0.01 ^b^
AI	0.15 ± 0.00 ^a^	0.15 ± 0.00 ^a^
TI	0.35 ± 0.00 ^a^	0.34 ± 0.00 ^b^
H/H	7.12 ± 0.04 ^a^	7.08 ± 0.04 ^a^
%	100.00 ± 0.00	100.00 ± 0.00

Values represent means of triplicate measurements ± standard deviation and are expressed as g FAME/100 g total FAME. The data were analysed using independent-sample *t*-tests, and within each row, different letters indicate statistical differences at *p* < 0.05. (FAME—fatty acid methyl ester, SFAs—saturated fatty acids. MUFAs—monounsaturated fatty acids, PUFAs—polyunsaturated fatty acids, RICR—Crăciunelu de Jos Italian Riesling GP, RICI—Ciumbrud Italian Riesling GP).

**Table 3:** Antiradical activity of the samples evaluated using the capacity of scavenging DPPH˙ and ABTS˙^+^, electron donation capacity using the FRAP, RP, and CUPRAC methods, and ferrous and cupric ions chelation capacity.

Sample/ Antioxidant Activity	DPPH	ABTS	CUPRAC	FRAP	RP	Ferrous Ion Chelation	Cupric Ion Chelation
	µM TE/mL Extract	mg GAE/mL Extract	mg GAE/mL Extract	mg GAE/mL Extract	mg GAE/mL Extract	µM EE/mL Extract	µm EE/mL Extract
RICR	81.16 ± 0.9 ^a^	3.23 ± 0.06 ^a^	0.89 ± 0.01 ^a^	1.54 ± 0.05 ^a^	1.14 ± 0.04 ^a^	0.23 ± 0.00 ^a^	0.41 ± 0.02 ^a^
RICI	88.61 ± 0.79 ^b^	3.00 ± 0.03 ^b^	1.08 ± 0.02 ^b^	1.81 ± 0.02 ^b^	1.11 ± 0.03 ^a^	0.22 ± 0.01 ^a^	0.34 ± 0.00 ^b^

Values represent means of triplicate measurements ± standard deviation and are expressed as μg ml^−1^ extract. Independent-sample *t*-tests were used to analyse the data, and within each row, different letters indicate statistical differences at *p* < 0.05. TE—Trolox equivalent, GAE—gallic acid equivalent, EE—EDTA equivalent; DPPH—2,2-diphenyl-1-picrylhydrazyl assay; ABTS—2,2′-azinobis-(3-ethylbenzthiazolin-6-sulfonic acid) radical-scavenging assay; CUPRAC—Cupric Reducing Antioxidant Capacity; FRAP—Ferric Reducing Antioxidant Potential; RP—Reducing Power; RICR—Italian Riesling cultivar from Crăciunelu de Jos vineyard; RICI—Italian Riesling cultivar from Ciumbrud vineyard.

## Data Availability

The original contributions presented in this study are included in the article. Further inquiries can be directed to the corresponding authors.

## Abbreviations

The following abbreviations are used in this manuscript:

GP	grape pomace
RICI	GP from Ciumbrud Italian Riesling grapes
RICR	GP from Crăciunelu de Jos Italian Riesling grapes
FTIR	Fourier transform infrared spectroscopy
HPLC-DAD-ESI MS	liquid chromatography–diode array detection–electrospray ionisation mass spectrometry
GC-FID	gas chromatography with flame ionisation detector
DPPH	2,2-diphenyl-1-picrylhydrazyl radical-scavenging capacity
ABTS	2,2′-azinobis-(3-ethylbenzthiazolin-6-sulfonic acid) radical-scavenging capacity
CUPRAC	cupric reducing antioxidant capacity
FRAP	ferric reducing antioxidant potential
RP	reducing power
TI	thrombogenicity index
AI	atherogenicity index
H/H	ratio between hypo- and hypercholesterolemic fatty acids
COX	calculated oxidizability
TPC	total polyphenol content
GAE	gallic acid equivalent
TE	Trolox equivalent
EE	EDTA equivalent
SFA	saturated fatty acid
UFA	unsaturated fatty acid
MUFA	monounsaturated fatty acid
PUFA	polyunsaturated fatty acid
